# Single-cell RNA sequencing reveals gene expression signatures of breast cancer-associated endothelial cells

**DOI:** 10.18632/oncotarget.23760

**Published:** 2017-12-29

**Authors:** Zhengda Sun, Chih-Yang Wang, Devon A. Lawson, Serena Kwek, Hugo Gonzalez Velozo, Mark Owyong, Ming-Derg Lai, Lawrence Fong, Mark Wilson, Hua Su, Zena Werb, Daniel L. Cooke

**Affiliations:** ^1^ Division of Neurointerventional Radiology, Department of Radiology and Biomedical Imaging, University of California, San Francisco, CA 94143, USA; ^2^ Department of Anatomy, University of California, San Francisco, CA 94143, USA; ^3^ Department of Physiology and Biophysics, University of California, Irvine, CA 92697, USA; ^4^ Division of Hematology and Oncology, Department of Medicine, University of California, San Francisco, CA 94143, USA; ^5^ Department of Biochemistry and Molecular Biology, Institute of Basic Medical Sciences, College of Medicine, National Cheng Kung University, Tainan 70101, Taiwan; ^6^ Center for Cerebrovascular Research, Department of Anesthesia and Perioperative Care, University of California, San Francisco, CA 94143, USA

**Keywords:** breast cancer, endothelial cell, single-cell RNA sequencing, extracellular matrix

## Abstract

Tumor endothelial cells (TEC) play an indispensible role in tumor growth and metastasis although much of the detailed mechanism still remains elusive. In this study we characterized and compared the global gene expression profiles of TECs and control ECs isolated from human breast cancerous tissues and reduction mammoplasty tissues respectively by single cell RNA sequencing (scRNA-seq). Based on the qualified scRNA-seq libraries that we made, we found that 1302 genes were differentially expressed between these two EC phenotypes. Both principal component analysis (PCA) and heat map-based hierarchical clustering separated the cancerous versus control ECs as two distinctive clusters, and MetaCore disease biomarker analysis indicated that these differentially expressed genes are highly correlated with breast neoplasm diseases. Gene Set Enrichment Analysis software (GSEA) enriched these genes to extracellular matrix (ECM) signal pathways and highlighted 127 ECM-associated genes. External validation verified some of these ECM-associated genes are not only generally overexpressed in various cancer tissues but also specifically overexpressed in colorectal cancer ECs and lymphoma ECs. In conclusion, our data demonstrated that ECM-associated genes play pivotal roles in breast cancer EC biology and some of them could serve as potential TEC biomarkers for various cancers.

## INTRODUCTION

Tumor endothelial cells (TEC) or tumor-derived EC, including both blood vessel origin and lymphatic origin, play an indispensible role in tumor growth and metastasis [1, 2]. The tumor environmental changes, such as hypoxia and chronic growth factor stimulation, could induce a series of endothelial dysfunctions including irregular diameters, fragility, leakiness and abnormal blood flow [3–6]. A series of *in vitro* studies indicated that the direct and indirect communications between TEC and tumor cells create and maintain a specific EC trait for the tumor niche and allows for the reciprocal growth factor exchange between endothelial and malignant cells that may also stimulate angiogenesis and neovascularization [7–12]. Although all these studies suggest the promising application of anti-angiogenic therapy for different kinds of tumors, the limited effects of this therapy in clinical trials suggest the functional and phenotypical heterogeneity of TEC from their normal counterpart [13–19]. For example, transforming growth factor beta (TGFβ) induces a spectrum of EC phenotypes with different functions that could underlie the plasticity and heterogeneity of the tumor vasculature [20]. All of these underscore the necessity of characterizing the heterogeneous gene expression profile of TEC.

A handful of studies have addressed the gene expression patterns of TEC compared with different control ECs by using two popular high-throughput gene expression technologies, microarray and serial analysis of gene expression (SAGE) [21–28]. Although these valuable studies have shed much light on characterizing gene expression patterns and finding potential biomarkers of TEC, both DNA microarray and SAGE need voluminous mRNA extracted from millions of ECs that is often difficult to attain given limitations of available surgical tissue. Furthermore, growing evidence illustrates that seemingly homogeneous cell populations can show considerable heterogeneous expression patterns because of multiple factors inside and outside the cells [29, 30]. The DNA microarray results from expression analyses of bulk tissue or large cell populations cannot reflect cell-to-cell variability and thus discover the specific cellular subtypes. A more complete picture of individual TEC functional condition under specific cancerous environments requires an assay that can analyze the whole gene expression profile or global transcriptome at the single-cell level [31–36].

The recent development of single-cell RNA sequencing (scRNA-seq), which combines single cell isolation techniques with RNA-seq, facilitates detecting the global transcriptome of thousands of isolated cells on single cell level by exhaustively quantifying the studied transcripts [37]. During the past few years, several groups have developed single-cell cDNA library development techniques [38, 39], including the “Smart-seq2” technology by Sandberg group that can generate quantitative and reproducible data from both single cells and small amounts of purified RNA [40]. Compared with other protocols, Smart-seq2 has improved reverse transcription, template switching and pre-amplification to increase both yield and length of cDNA libraries generated from individual cells [41].

In this study, we address the feasibility of using the Smart-seq2 scRNA-seq technique to profile the transcriptome of single ECs isolated from human breast cancer tissues and build up a bioinformatics analysis flowchart for acquired libraries based on a series of state-of-the-art computational biology toolsets.

## RESULTS

### scRNA-seq libraries making from breast tissue isolated ECs

**Figure 1:**
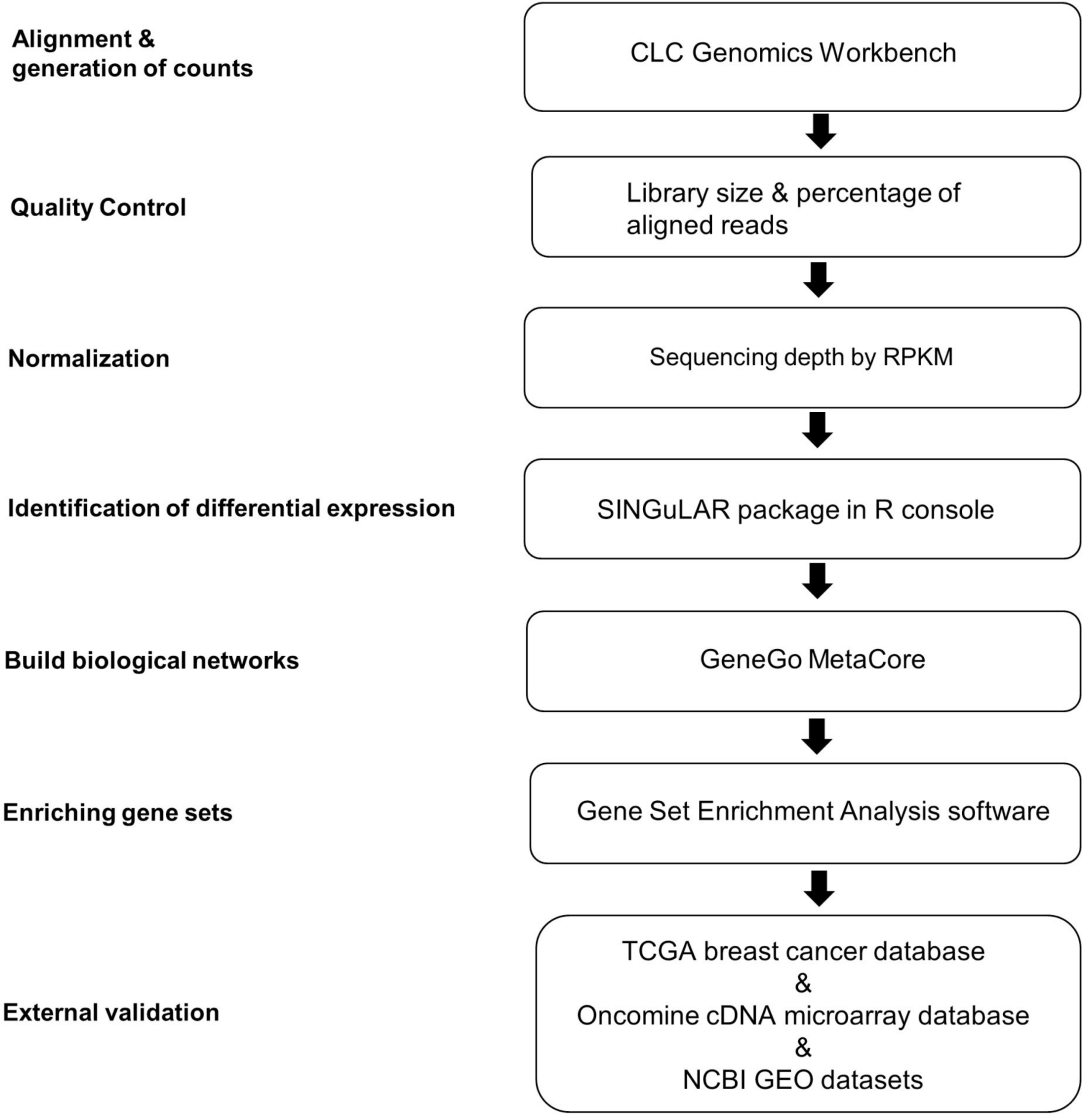
Analytical strategies of scRNA-seq library database A flow chart of the steps in analysis of scRNA-seq data from single ECs is shown. The analysis steps are listed on the left. The computational biology toolsets used for the steps are listed in the boxes. RPKM, reads per kilobase of transcript per million mapped reads; TCGA, The Cancer Genome Atlas.

**Figure 2:**
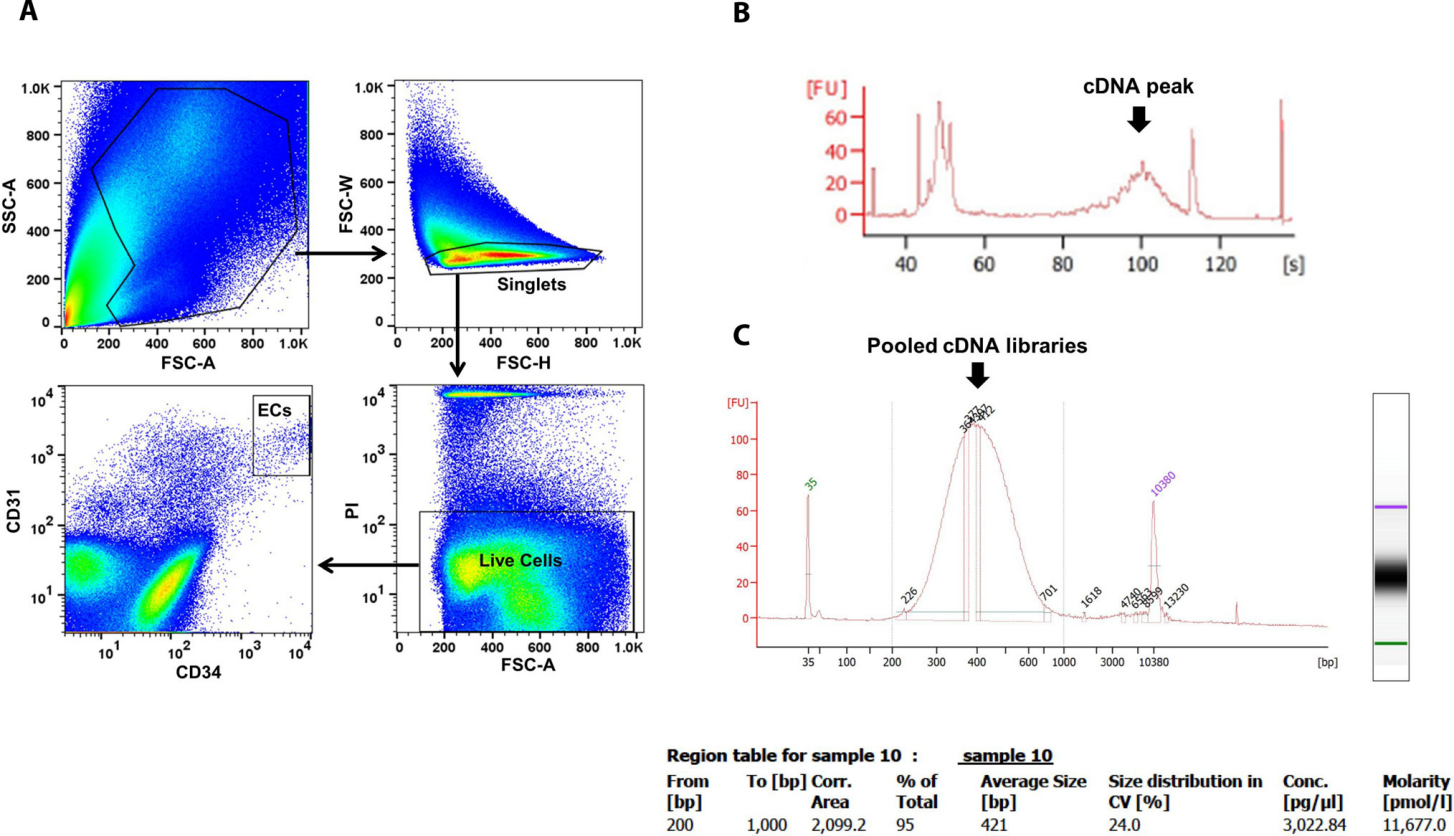
EC sorting strategy on FACS and single-cell cDNA libraries quality control by Bioanalyzer ECs were sorted by CD31 and CD34 markers on viable single cells identified by PI (**A**). Representative Bioanalyzer electropherograms show an obvious cDNA peak at around 1 kb after cDNA pre-amplification PCR of one single EC (**B**), and the correct size and reasonable concentration of pooled single EC cDNA libraries (**C**). PI, propidium iodide.

The flowchart of scRNA-seq library development and analysis is shown in Figure 1. To ensure that the isolated ECs were viable for library making, only propidium iodide (PI)-negative single cells were gated for further EC markers sorting (Figure 2A). The final percentage for CD31 and CD34 double-positive ECs was less than 1% (0.2% in Figure 2A). cDNA pre-amplification PCR could only give obvious cDNA peaks on the Bioanalyzer by ≥22 cycles that is higher than the recommended 18 cycles previously published [40] (Figure 2B). This may also indicate the difficulty of making single-cell cDNA libraries from patient samples that have been stored for an extended period. The Bioanalyzer figure of pooled cDNA libraries after adaptor PCR amplification showed the correct size (450 bps) and reasonable concentration (∼10nM) that suggested the acceptable quality of the cDNA libraries for further sequencing (Figure 2C).

### Library quality control and screening

280 viable ECs were isolated from patient tissues, including 88 cells from two breast cancer samples and 192 cells from four reduction mammoplasty samples, and their transcriptomes were sequenced. Quality control analysis of scRNA-seq library indicated that only 146 (52%) libraries had more than one million reads including 74 cancerous EC libraries (84%) and 72 normal EC libraries (38%). This different library making efficiency between cancerous and control ECs (84% versus 38%) suggests that it might be harder to isolate high quality viable ECs from normal breast tissue than cancerous breast tissue, partly because of the lower EC component in control tissue. The percentages of reads aligned to genome are 59 ± 9.3% and 52 ± 12.2% for cancerous and control ECs, respectively. 6,865 genes were detected by the standard of RPKM>1 from these 146 EC cDNA libraries and only these genes in the libraries with more than one million reads were selected for further computational biology analyses.

### Principal component analysis separates the cancerous and normal ECs into two clusters

**Figure 3:**
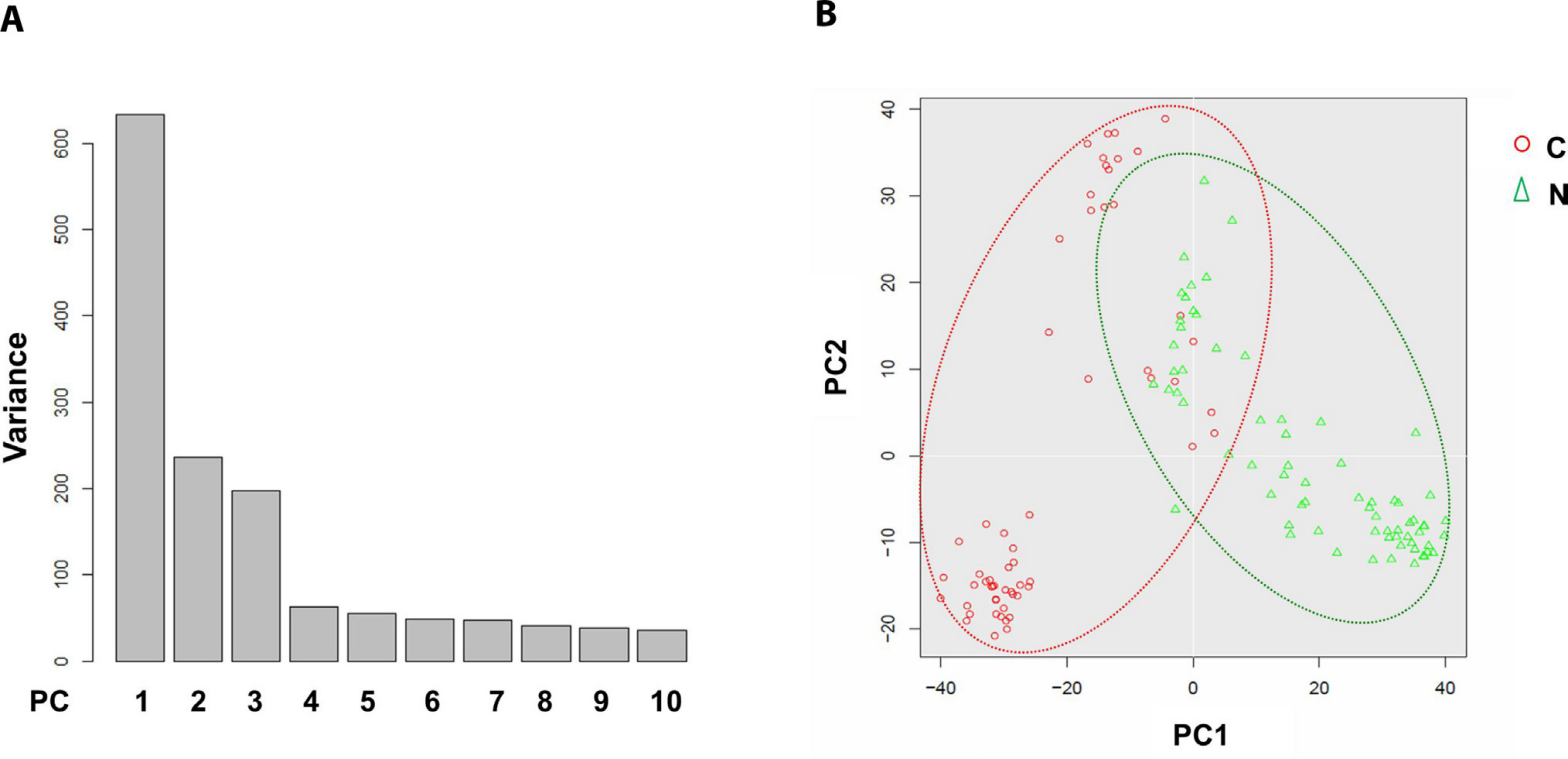
Principal component analysis of cancerous versus control EC libraries PCA screen plot indicates that PC1-3 could well explain the whole variance of the database (**A**). 2D PCA plot shows two distinct clusters along the PC1 axis that correspond to the cancerous ECs (red circle, C) and control ECs (green triangle, N) with 8 cancerous ECs overlapping into the control EC cluster (**B**).

**Figure 4:**
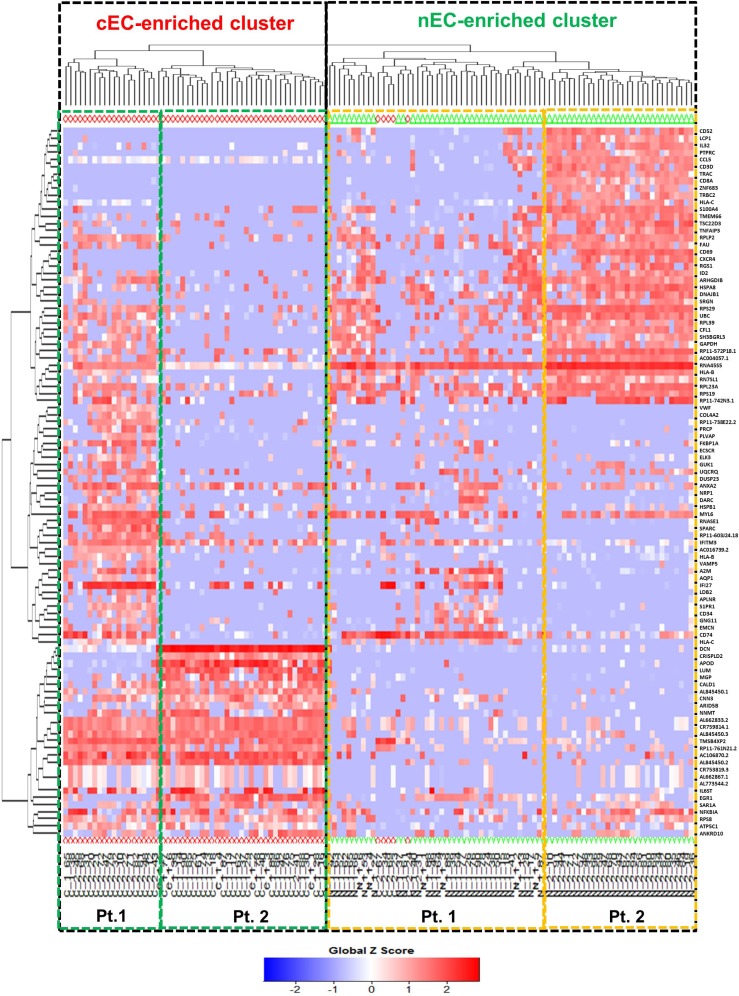
Heat map-based hierarchical clustering separates cancerous versus control ECs Cancerous ECs (cEC, red diamond) and control ECs (nEC, green triangle) are separated by heat map-based unsupervised hierarchical clustering produced from their gene expression profile of the top 100 genes with highest score PC1-3. The two clusters are highlighted by two black dot line boxes. Only 5 cancerous ECs clustered together with control ECs. Under each cluster, the cells are sub-grouped by their patient of origin.

To address the differences of transcriptome profile between the cancerous ECs and control ECs, unsupervised principal component analysis (PCA) was first used to study the potential clusters of these ECs based on the detected genes. The PCA screening plot visually indicated that the first three principal components (PC1-3) could well explain the whole variance of the database with PC1 itself explaining 29.8% of total variance (Figure 3A). 2D PCA plot also showed that PC1 and 2 separated the cancerous versus control ECs as two distinctive clusters with eight cancerous ECs overlapping into the control EC cluster (Figure 3B). This PCA result indicated that these two phenotypes of ECs could be separated by their transcriptome profile by unsupervised clustering. The top 100 genes that had the highest sums of score PC1-3 were also used to produce the heat map-based unsupervised hierarchical clustering analysis. The developed heat map and dendrogram also separated these two EC phenotypes into two distinctive clusters with only 5 overlapped cells (Figure 4). Most of the ECs originating from different patients clustered together in both cancerous and control EC clusters and developed subgroups of ECs. This may indicate the subtle gene expression differences exist between patients that might belong to different breast pathological types of breast cancer or benign breast hyperplasia. ANOVA showed that 82 of these 100 genes had significantly different expression levels between cancerous and control ECs (*p <* 0.05)

### Disease biomarker analysis verifies breast cancer-correlated gene expression profile

**Figure 5:**
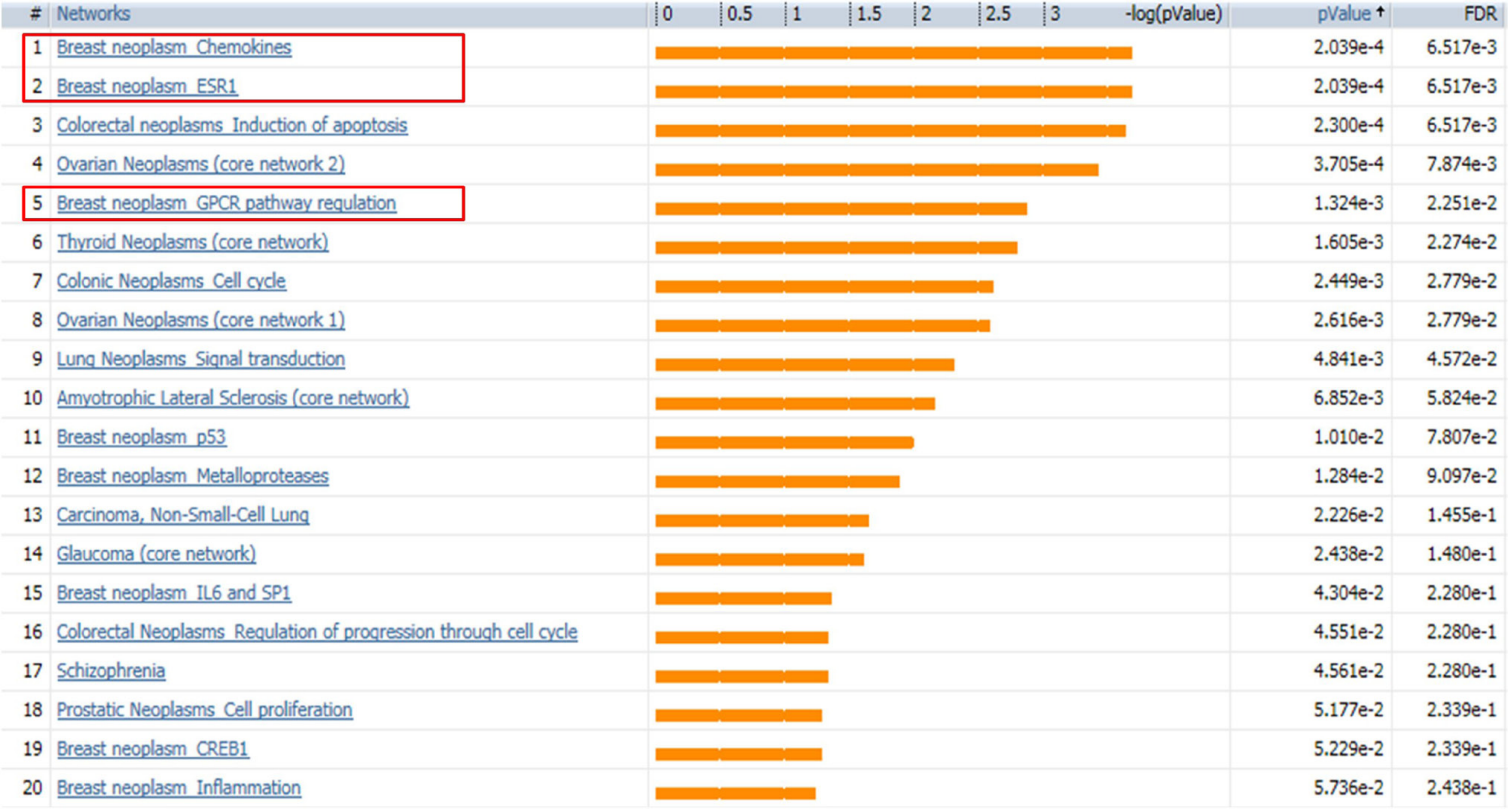
MetaCore disease biomarker analysis indicates the differentially expressed genes are correlated with breast cancer Genetic co-morbidity analysis indicated three of the top five disease annotations are breast neoplasm chemokines, breast neoplasm ESR1 and breast neoplasm GPCR pathway regulation. Orange bars represent the significance of difference by negative logical *p* value. *p* values and FDR values are listed on the right.

**Figure 6:**
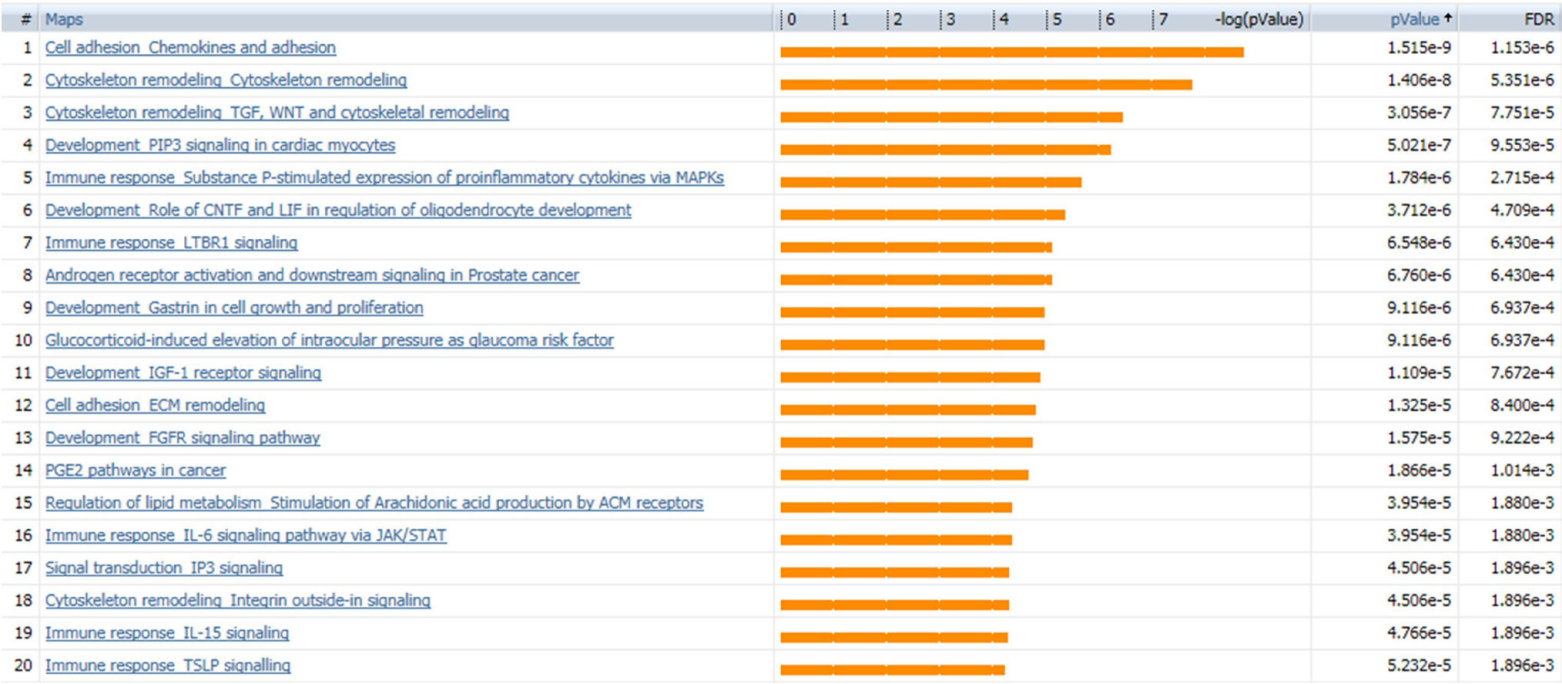
MetaCore pathway analysis suggests dozens of signaling pathways Pathway analysis by MetaCore indicated that the differentially expressed genes are highly correlated with dozens of signal pathways; the 20 most significant pathways are listed. Orange bars represent the significance of difference by negative logical *p* value. *p* values and FDR values are listed on the right.

SINGuLAR software identified 1,302 genes out of the total 6,865 genes were differentially expressed between cancerous and control ECs by the standard of *p <* 0.05 and expression fold change >4. MetaCore disease biomarker analysis indicated that, among the first 20 disease annotations, three of the top five were breast neoplasm chemokines (*p* value = 2.04 × 10^–4^ and FDR = 6.52x10^–3^), breast neoplasm estrogen receptor 1 (ESR1, *p* value = 2.04 × 10^–4^ and FDR = 6.52 × 10^–3^) and breast neoplasm G-protein coupled receptor (GPCR) pathway regulation (*p* value = 1.32 × 10^–3^ and FDR = 2.25 × 10^–2^) (Figure 5). This suggested that these differentially expressed genes identified by this scRNA-seq database are highly correlated with the breast neoplasm diseases and could represent the characteristic gene expression profile of breast cancer ECs to some extend. In addition, MetaCore pathway analysis indicated that these differentially expressed genes were also highly correlated with dozens of signal pathways like cell adhesion, cytoskeleton remodeling and immune response (Figure 6).

### Extracellular matrix related pathways are enriched by GSEA

**Figure 7:**
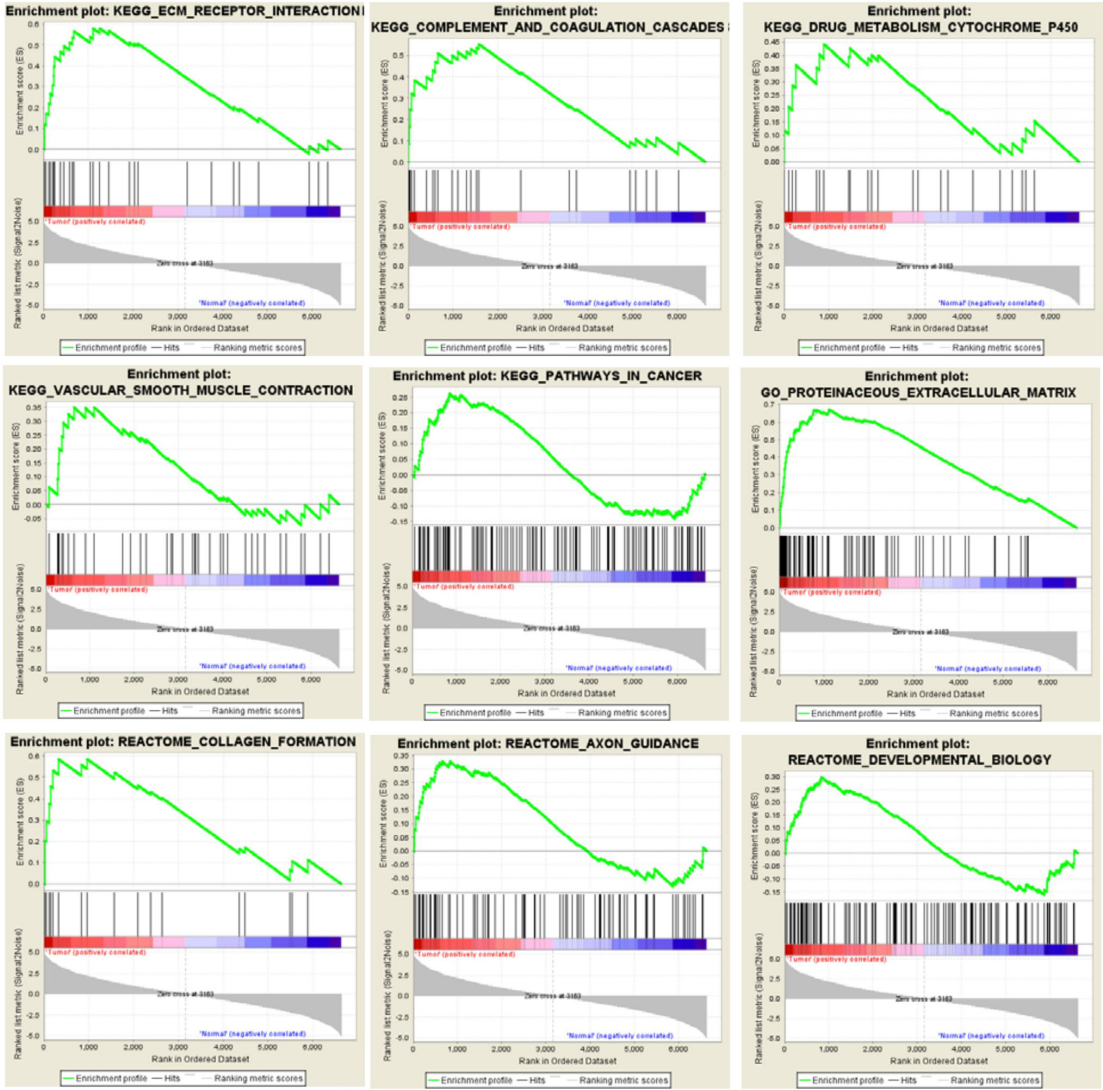
GSEA enriches differentially expressed genes into multiple functional gene networks GSEA results show that differentially expressed gene set in cancerous ECs are enriched for some EC-associated functional gene networks as well as some novel signaling pathways.

**Figure 8:**
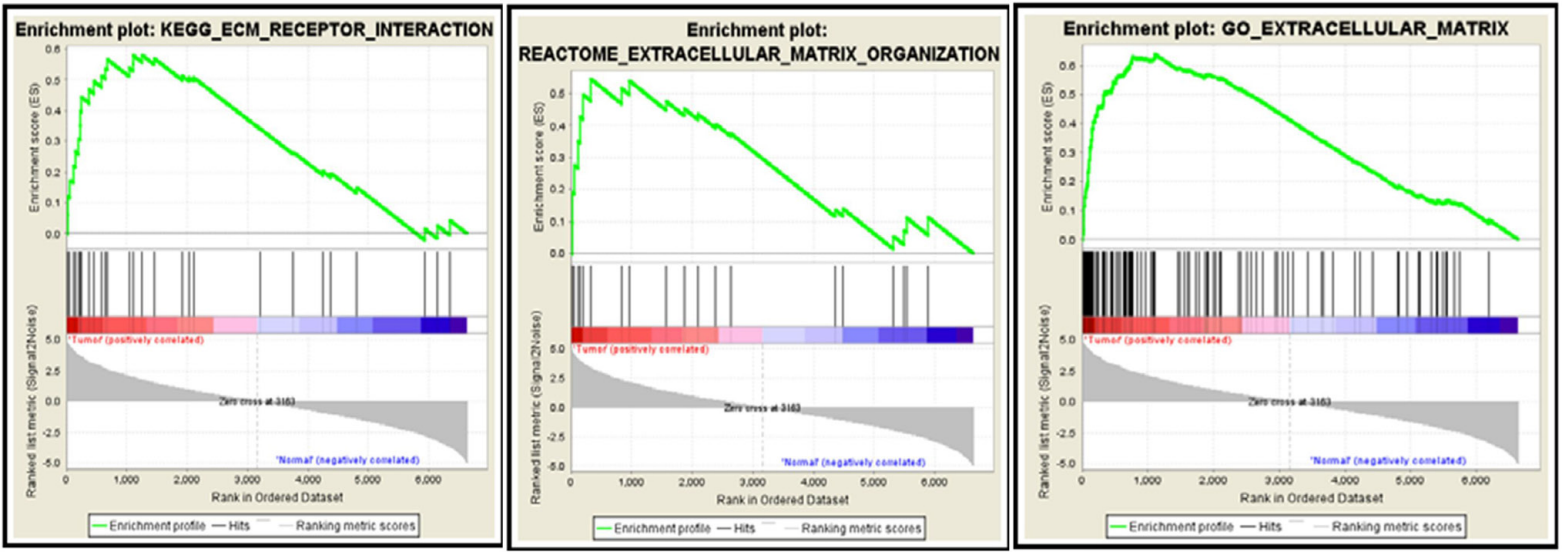
GSEA databases enrich the differentially expressed genes to ECM-associated pathways 30 genes by KEGG database are enriched for ECM receptor interaction pathway (Left), 20 genes by Reactome database for ECM organization pathway (Middle) and 116 genes by Gene Ontology database for ECM-related pathway (Right). Red bar and blue bars represent upregulated and downregulated genes, respectively.

**Figure 9:**
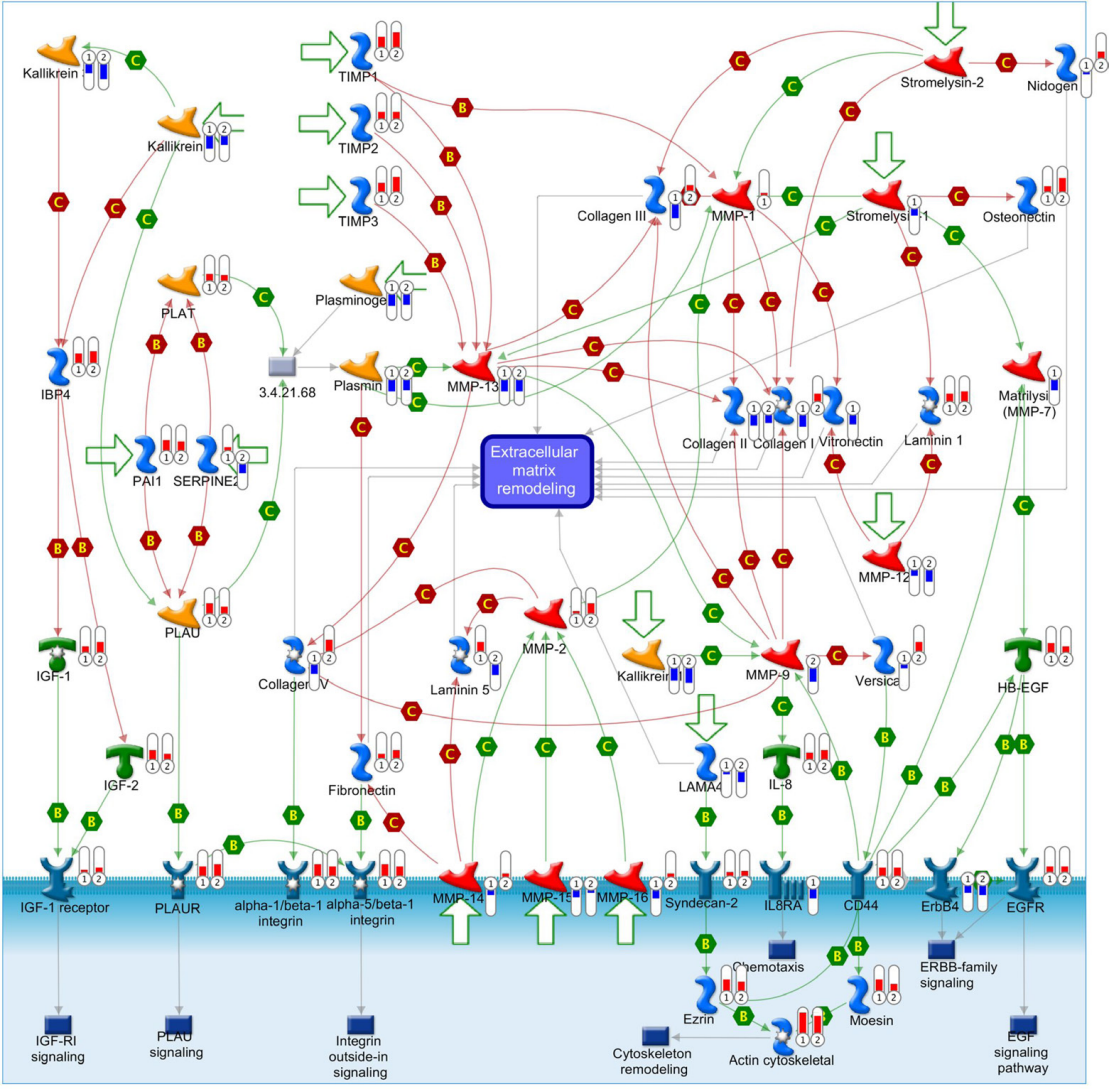
ECM-associated genes are differentially expressed in cell adhesion ECM remodeling gene network This comprehensive picture shows gene expression changes and crosstalk of pathways in the gene network of cell adhesion ECM remodeling. The differentially expressed genes detected in our database are labeled with thermometers that indicate gene expression changes in TECs (1) and control ECs (2). Upward thermometers with red color reflect up-regulated expression and downward thermometers with blue color reflect down-regulated expression. There are more ECM-associated genes were highly expressed in TECs than control ECs. “B” and “C” mean binding and cleavage respectively. A legend explaining the symbols used by MetaCore is provided at http://portal.genego.com/legends/legend_6.png.

GSEA results showed that the differentially expressed gene set was enriched for some functional gene networks that are clearly associated with ECs, such as extracellular matrix (ECM) metabolism, vascular smooth muscle contraction and collagen formation, as well as some novel signal pathways such as complement and coagulation, drug metabolism, cancer pathways and axon guidance (Figure 7). What was most notable, three classic Gene Set Enrichment Analysis (GSEA) databases, Kyoto Encyclopedia of Genes and Genomes (KEGG), Reactome and Gene Ontology (GO), all indicated that the differentially expressed gene set was enriched for the ECM-related pathways (Figure 8). Specifically, 30 genes were enriched by KEGG database for ECM receptor interaction pathway, 20 genes were enriched by Reactome database for ECM organization pathway, and 116 genes were enriched by Gene Ontology database for ECM-related pathway. Altogether, these three databases enriched 127 genes that are related to ECM. This suggested the pivotal roles of ECM metabolism in the EC phenotype of breast cancer. Moreover, when Metacore thermometer figure was used to show the expression levels of GSEA enriched genes in one gene network of ECM remodeling, the thermometer reading levels indicated that many ECM associated genes in this network were differentially expressed and there are more genes had higher expression levels in cancerous ECs than in control ECs (Figure 9).

### Gene external validation by TCGA breast cancer database

**Figure 10:**
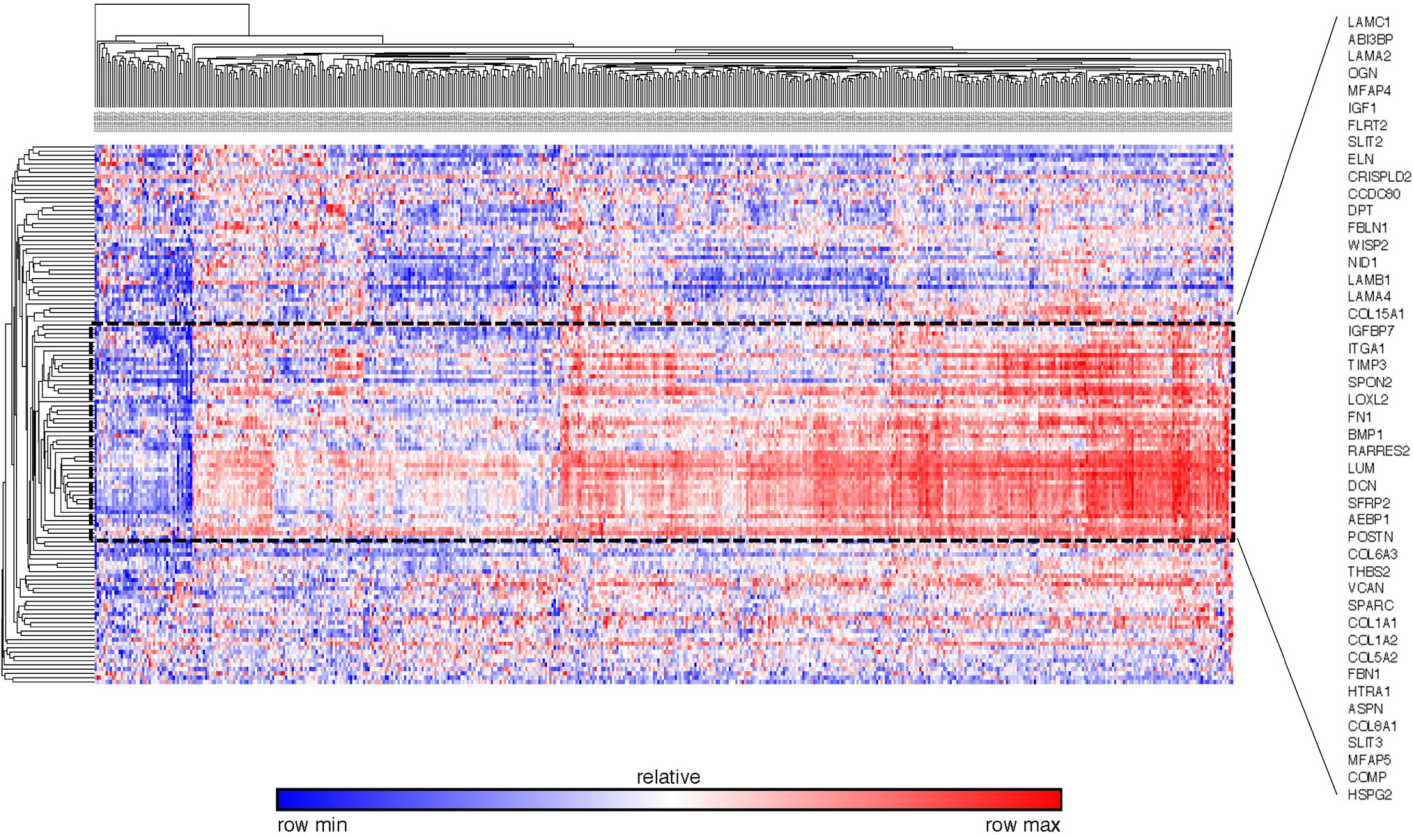
External validation of ECM-associated genes by the TCGA breast cancer database The heat map and hierarchial clustering presented the ECM-associated gene expression level of different breast patients in TCGA database The patients and genes are listed along the X and Y axles respectively. The black dot line highlights the 46 genes that separated the patients in the database into visual subgroups.

**Figure 11:**
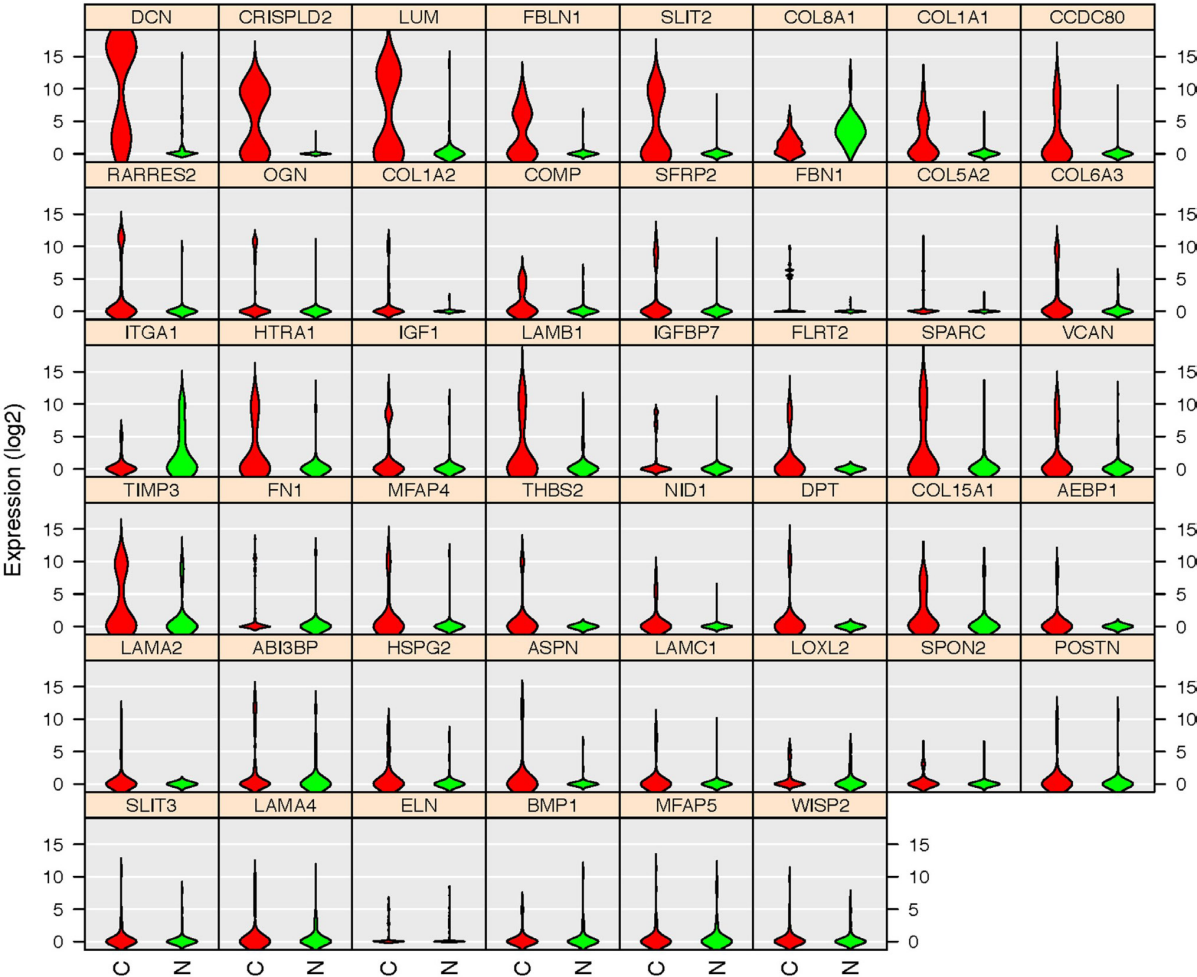
Gene expression patterns of the 46 TCGA database validated genes Violin plots indicated that 43 genes are upregulated in cancerous ECs (Red) compared with control ECs (Green) in our database. Only three genes (COL8A1, ITGA1 and MFAP5) were downregulated. The gene name is indicated on top of each violin plot and the value on Y-axis represents the gene expression level in the binary logarithm (log2) value.

To validate the differentially expressed gene set found by our database, we extracted the expression pattern of the GSEA-enriched ECM-associated genes in the TCGA breast cancer patient database through Oncomine. The heat map and hierarchical clustering indicated that 46 genes clustered the patients in the database into visual subgroups (Figure 10). The gene expression pattern also showed that 43 out of these 46 validated genes were upregulated and only three, collagen type VIII alpha 1 Chain (COL8A1), integrin subunit alpha 1 (ITGA1) and microfibril associated protein 5 (MFAP5), were downregulated in these breast cancer ECs compared with the control ECs (Figure 11).

### ECM associated genes are upregulated in a variety of cancers

**Figure 12:**
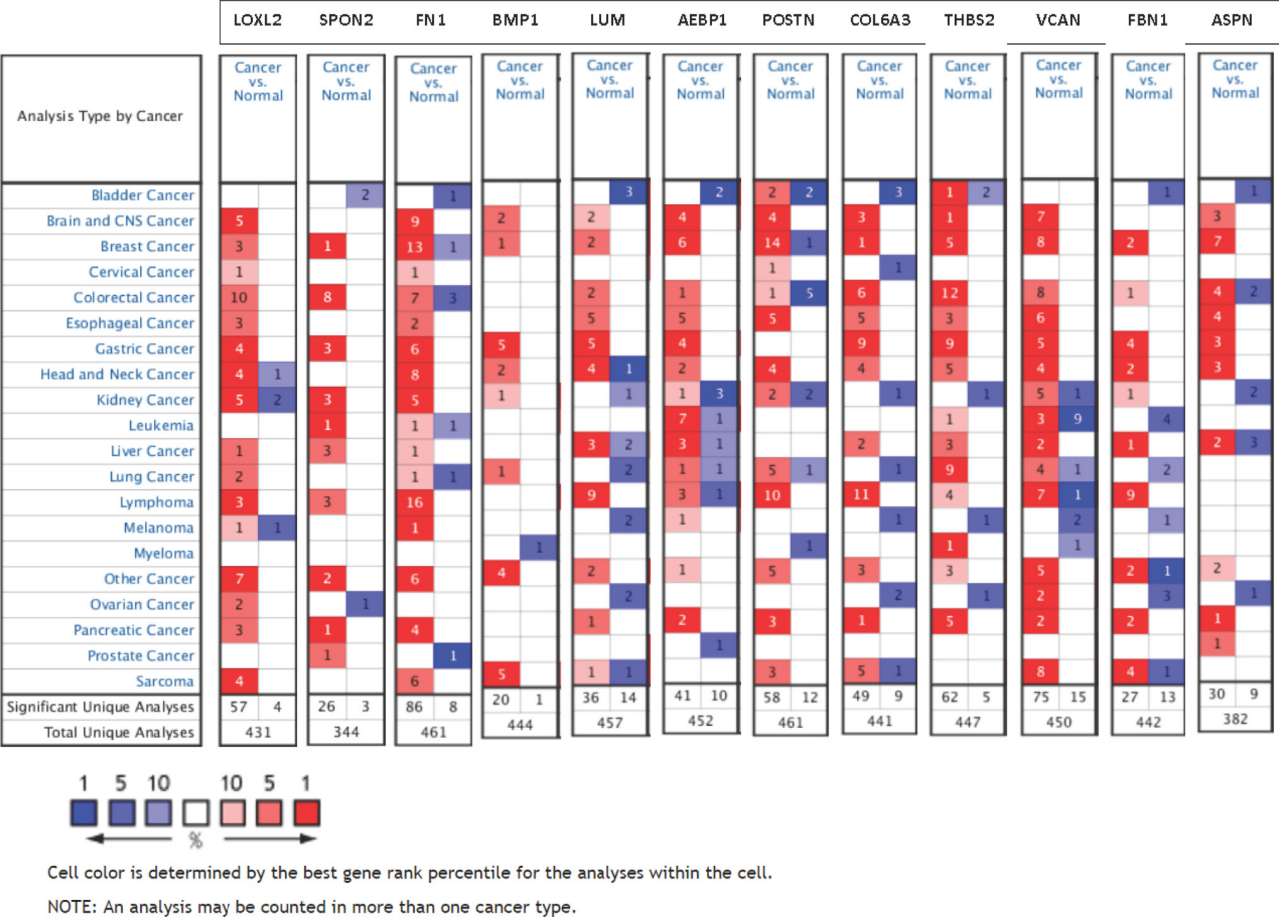
Expression of 12 oncogenic ECM-associated genes in different cancer types The number of datasets that had significant mRNA overexpression (left column, red) and underexpression (right column, blue) of 12 specified genes are compared in 20 cancer types versus normal tissue. The color gradient correlates with decreasing gene rank percentile. The search criteria threshold was set at *p*-value <0.0001 with fold change >2.0 and gene rank percentile <10% for screening microarray datasets of cancer versus normal cases.

To address if these 46 ECM-associated genes that were validated by TCGA breast cancer database are also important in other types of cancer, we extracted transcript expression data of these genes for 20 common cancer types from the Oncomine cDNA microarray database. 12 genes showed overall overexpression in tumors vs. normal tissues by the majority of the datasets (Figure 12), including Lysyl oxidase-like-2 (LOXL2, 57 vs. 4), Spondin-2 (SPON2, 26 vs. 3), fibronectin-1 (FN1, 86 vs. 8), bone morphogenetic protein-1 (BMP1, 20 vs. 1), Lumican (LUM, 36 vs. 14), Adipocyte enhancer-binding protein-1 (AEBP1, 41 vs. 10), Periostin (POSTN, 58 vs. 12), Collagen VI α3 (COL6A3, 49 vs. 9), Thrombospondin-2 (THBS2, 62 vs. 5), Versican (VCAN, 75 vs.15), Fibrillin-1 (FBN1, 27 vs. 13) and Asporin (ASPN, 30 vs. 9). These data suggest these 12 ECM-associated genes that may play oncogenic function in different cancer types and are not limited to breast cancer. Among these 20 cancers, colorectal cancer and lymphoma tissues showed remarkably high expression of these 12 genes (Figure 12).

We also investigated whether the mutation status of these 12 ECM related genes are favored during oncogenesis. We used meta-analysis to study the ECM gene mutation status in TCGA and other public breast cancer databases via cBioPortal platform [42]. Strikingly, there was an extremely low frequency of alterations disrupting these ECM function. In total 4063 breast tumor samples, we found only 239 point mutations were recorded at these ECM genes in these tumors (all frequencies are less than 1% except COL6A3 was 4%). These ECM mutation frequencies are thus considered very low, as high-frequency mutations are typically described as being over 20% and intermediate-frequency mutations between 2 and 20% [43]. Thus, the lack of large-scale genomic aberrations and the non-synonymous mutation frequency of 0.1–4% make it unlikely that alterations of these ECM genes impact the cancer cell phenotype (Supplementary Figure 1).

### ECM genes are highly expressed in TEC from other cancers

**Figure 13:**
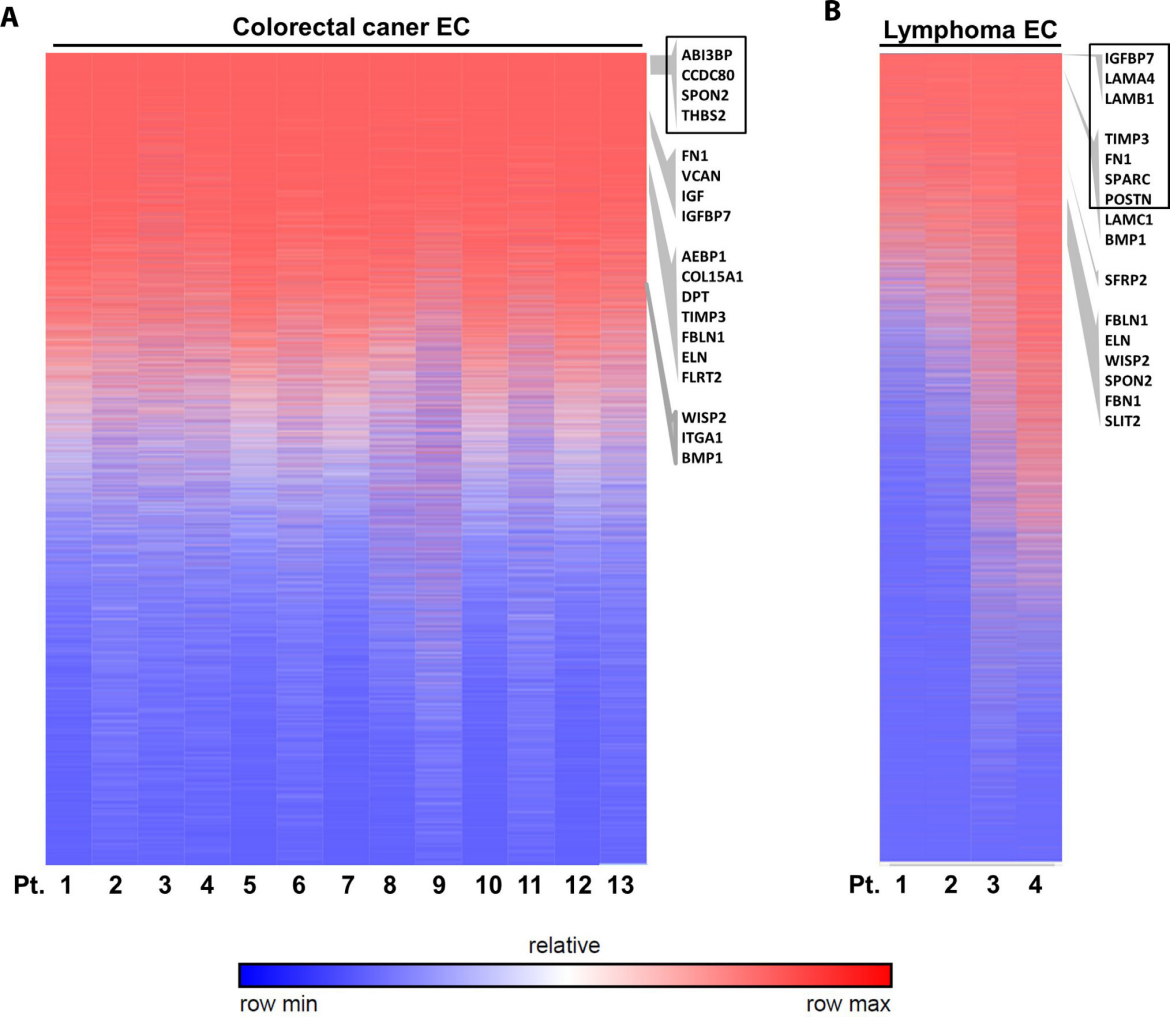
Validation of ECM-associated genes in other cancer ECs Analysis of published microarray data of colorectal cancer ECs (**A**) and lymphoma ECs (**B**) indicated the high expression of some ECM associated genes. The heat map shows genes rankings ordered from highest to lowest for raw expression values across different patient samples. GSEA enriched ECM genes are listed on the right according to their ranking of expression level. Genes ranked in top 2000 are highlighted by black box.

To address whether these validated genes are also differentially expressed in TECs from other cancer types, we go further to investigate their expression levels in colorectal cancer ECs and lymphoma ECs. Our data indicated that some of these genes specifically had high expression level in TECs from these two cancer types by analyzing available NCBI GEO database. Heat map of gene expression ranking indicated that these ECM associated genes were highly expressed in the colorectal cancer ECs (Figure 13A), especially with 4 genes ranked in the top 2000 genes out of over 44,000 detected microarray genes, i.e. ABI Family Member 3 Binding Protein (ABI3BP, No. 544), Coiled-Coil Domain Containing 80 (CCDC80, No.1378), SPON2 (No.1490) and THBS2 (No. 1883). Similarly in the lymphoma ECs, 7 genes ranked in the top 2000 genes, i.e., insulin like growth factor binding protein 7 (IGFBP7, No.83), laminin subunit alpha 4 (LAMA4, No.106), laminin subunit beta 1 (LAMB1, No.188), tissue inhibitor of metalloproteinase 3 (TIMP3, No.509), FN1 (No.1025), secreted protein acidic and cysteine rich (SPARC, No.1146) and POSTN (No.1890) (Figure 13B). These data suggested these ECM associated genes as possible universal TEC markers not only in breast cancer but also in colorectal cancer and lymphoma.

## DISCUSSION

In this study, we have shown the feasibility of developing scRNA-seq libraries from viable single ECs isolated from overnight stored clinical tissue. This is also the first report of the signature gene expression profile of breast cancer-derived ECs on a single-cell level. Our study showed that ECM-associated genes play a key role in tumor-derived ECs and identified some of these genes could serve as universal TEC biomarkers.

Although fresh tissue samples are always recommended for RNA sequencing to ensure the quality of RNA especially mRNA [40], practically, it is difficult to get such fresh tissues all the time because of the unpredictability of the surgical schedule and/or the accessibility of experiment resources. The detectable gene numbers in the transcriptome by scRNA-seq varies among studies because of different cell type, cell size, sequencing depth, etc. [44]. Furthermore, although there is still no standard detectable gene number thus far, a common accepted standard is that 1 million reads per single cell library could detect >90% genes that has FPKM>1 [44]. In this study, using the sub-optimal breast cancerous and control reduction mammoplasty tissue, we found that such qualified single EC libraries can be made for transcriptome characterization even by relative “shallow” depth sequencing, though at a relatively lower percentage of read alignment to genome and a lower number of detected genes (about 7,000). We suggest several points are important for making cDNA libraries from sub-optimal tissues for scRNA-seq that echo the technique details emphasized in a previous publication [40, 44], such as identifying viable cells by viability markers as PI on FACS, increasing the cycle numbers to >20 for cDNA pre-amplification PCR and quality control of cDNA libraries and sequencing database. Our work indicates that sub-optimal clinical tissue, as kept overnight in storage buffer, may still be used to isolate single cells such as ECs that can be used for scRNA-seq. This may expand the usage scope for this state-of-the-art technique to study the transcriptome under different disease conditions.

In this scRNA-seq study, our results highlighted the important role of ECM in breast cancer EC biology. As a major component of the cancer local microenvironment, ECM has been reported to play important roles in cancer development [45–49]. The tightly controlled properties of ECM during embryonic development and tissue homeostasis are deregulated and disorganized in cancers. Besides directly acting on cancer progression by inducing cellular transformation and metastasis, ECM also facilitates development of a tumorigenic microenvironment by manipulating tumor-associated angiogenesis and inflammation. Therefore, it is expected that some ECM-associated genes may provide potential therapeutic targeting the tumor niche for treatment.

Overexpression of ECM-associated genes has also consistently been reported in previous studies on isolated TECs. In the colorectal cancer EC study of St. Croix *et al.* [21], seven out of the top 25 most differentially expressed biomarkers encode proteins involved in ECM formation or remodeling. Parker *et al.* found several ECM regulating genes overexpressed in isolated breast cancer ECs including osteonectin, matrix metallopeptidase 9 (MMP9), and tissue inhibitor of metalloproteinase 1 (TIMP1) [23]. Madden *et al.* also found several ECM architecture regulating genes among the gene products identified as glioma endothelial markers, including heparan sulfate proteoglycan 2, several type IV collagen transcript variants, and matrix metallopeptidase 14 (MMP14) [50]. Similar to our study, Bhati *et al.* found 1176 genes were differentially expressed in human luminal-A breast tumors compared with normal vascular cells with the extracellular matrix gene ontology category was increased [24]. All these findings suggest that ECM changing is an essential component of alterations in tumor endothelium.

Although all of these 12 ECM-associated genes have been reported to be involved in the different stages of cancers like tumorigenesis, invasion and metastasis in different cancer types and were used as diagnostic biomarker or prognostic indicator, only very limited studies have addressed their expression level in TEC specifically. LOXL2-neutralizing antibody, AB0023, was reported to inhibit angiogenesis in part by affecting VEGF signaling in ECs and simtuzumab, a humanized version of AB0023, is currently being evaluated in clinical trials for the treatment of advanced solid tumors [51]. BMP-1 overexpression in ECs was shown to be restricted to areas of tumor angiogenesis *in vivo* [52]. Recently, the EC-secreted fibronectin extra domain A (EDA) was demonstrated to promote the vasculogenesis, tumorigenesis and metastasis of colorectal carcinomas (CRCs) [53]. Thus far, no studies have reported the gene expression changes in SPON2, FN1, LUM, AEBP1, POSTN, COL6A3, THBS2, VCAN, FBN1, ASPN in TEC.

What is noteworthy is that, NCBI GEO gene expression database of TECs in colorectal cancer and lymphoma also verified the specific high expression of 7 out of these 12 ECM associated genes in TECs from colorectal cancer and lymphoma, i.e. SPON2, BMP-1, FN1, POSTN, THBS2, VCAN and AEBP1. These data suggested these ECM associated genes as possible universal TEC markers not only in breast cancer but also in colon cancer, lymphoma and other cancers. Because of the limitation of sample size in this study, further analysis of scRNA-seq database from more TECs in different cancer tissues and according verification experiments are needed to justify these new biomarkers.

## METHODS

### Origin of tissue and single viable EC harvest

Both breast cancer and reduction mammoplasty tissues were overnight-shipped from Cooperative Human Tissue Network (CHTN), a program funded by the National Cancer Institute. Single viable ECs were isolated following the protocol of van Beijnum *et al.* with minor modification [54]. Briefly, about 0.5 g of tissue was minced using surgical blades. Mashed tissues were then enzymatically digested for 1 hour in 10 ml digestion buffer (0.1% collagenase II 9 ml, 2.5 U/ml dispase 1ml and 0.1% DNase 75 ml) in a 37°C water bath under continuous agitation. Dissociated single cells were separated by sieving the samples through 100 mm cell strainer and 400 x g centrifugation. The re-suspended cells were then stained with two EC surface markers, CD31 Alexa647 (BD Biosciences 561654) and anti-CD34 PE-Cy7 (BioLegend 343516), and cell viability marker Propidium iodide (PI, Abcam, ab14083). After removing red blood cells by ACK lysis buffer (Life Technologies, A1049201), viable single ECs were sorted on a FACS Aria II (BD Biosciences, San Jose, CA) with 100 nm nozzle by the standard of CD31 and CD34 double positive single cells with negative PI staining. Each single EC was sorted directly into cell lysis buffer on a 96-well plate (Eppendoff, Hauppauge, NY).

### Single-cell RNA sequencing

Single-cell RNA isolation, reverse transcription and cDNA pre-amplification were performed following the Smart-Seq2 protocol of Picelli *et al* [40]. cDNA library preparation was performed following the Fluidigm C1 Protocol (100-7168 I1). Quantification of cDNA libraries was performed using Quant-iT PicoGreen dsDNA Assay Kits (Thermo Fisher Scientific, P11495) and high-sensitivity DNA chips (Agilent 5067-4626). The pooled libraries were cleaned up following the Double-sided Size Selection Protocol in KAPA Hyper Prep Kit (Kapa Biosystems, KR0961). To minimize the systemic errors, all libraries were loaded on the two lanes of one Rapid mode v2 SR1x100 flow cell and sequenced by Illumina HiSeq 2500 resulting in a calculated depth of ∼1.0 million reads per cell (Illumina Rapid SR Cluster Kit v2 GD-402-4002 and Rapid SBS kit v2 FC-402-4022).

### Processing of scRNA-seq library dataset

The analytical strategies of scRNA-seq library database are shown in Figure 1. After de-multiplexing sequencing data to FASTQ files, the libraries that passed quality control assessment performed by FASTQC were further aligned to an indexed hg19 RefSeq genome using transcriptome analysis toolkits CLC Genomics Workbench (Qiagen, v6.0.4, CLCbio, Arhus, Denmark). Reads per kilobase of transcript per million mapped reads (RPKM) values of detected genes in each library were calculated for subsequent analyses. RPKM value is calculated by dividing the read counts of a certain gene in a library by the “per million” scaling factor and the length of the gene in kilobases sequentially to normalize both sequencing depth and gene length. The “per million” scaling factor is calculated by dividing the total reads number in the library by 1,000,000. For quality control filtering, only genes with a RPKM>1 in at least one sample were used for downstream computational biology analyses. Differentially expressed genes identification by one-way ANOVA, principal component analysis (PCA) and hierarchical clustering based on heat map were all performed in the Fluidigm SINGuLAR Analysis Toolset 2.0 R package after removing the outliers. A gene is considered to be differentially expressed when a probability *P* value <0.05 (with FDR correction) and expression fold change >4 between cancerous ECs versus control ECs. Genes are clustered on the basis of Pearson correlation and samples are clustered on the basis of a Euclidian distance matrix with complete linkage.

### Further computational biology analyses

The differentially expressed gene set was input into pathway analysis package GeneGo MetaCore (https://portal.genego.com/) to build biological networks and list the associated biological processes and diseases. A *p*-value of 0.05 was used as a cut off to determine significant enrichment of a pathway or annotated gene grouping present in the Metacore database. Gene Set Enrichment Analysis software (https://software.broadinstitute.org/software/cprg/?q=node/14) was also used to enrich gene sets or groups from these differentially expressed genes that share common biological function, chromosomal location, or regulation [55]. As a freely available software package together with an initial database of 1,325 biologically defined gene sets, GSEA is a computational method that determines whether a pre-defined set of genes shows statistically significant, concordant differences between two biological states. GSEA-enriched genes were then externally validated in The Cancer Genome Atlas (TCGA) breast cancer database and the web-based Oncomine cDNA microarray database (http://www.oncomine.org) were also used to identify the clinical significance and expression level of these TCGA-validated genes in other cancer types. Published microarray data sets of TECs from 13 colon cancer patients (GSE89287) and 4 lymphoma patients (GSE8852) were downloaded from Gene Expression Omnibus (GEO). Microarray gene expression values were then calculated by global median normalization.

### Data availability

The datasets generated during and/or analysed during the current study are available from the corresponding authors on reasonable request.

### Ethical approval statement

All the methods used in this study were carried out in accordance with the relevant guidelines and regulations of University of California San Francisco.

## SUPPLEMENTARY MATERIALS FIGURE


