# Supplementary material for: Single-cell RNA sequencing reveals gene expression signatures of breast cancer-associated endothelial cells
Source: Oncotarget. 2017 Dec 29;9(13):10945–61. doi: 10.18632/oncotarget.23760 (PMC5834272; doi:10.18632/oncotarget.23760)
Supplement: Supplementary file 1 [file oncotarget-09-10945-s001.pdf]

Single-cell RNA sequencing reveals gene expression signatures of breast cancer-associated endothelial cells

SUPPLEMENTARY MATERIALS

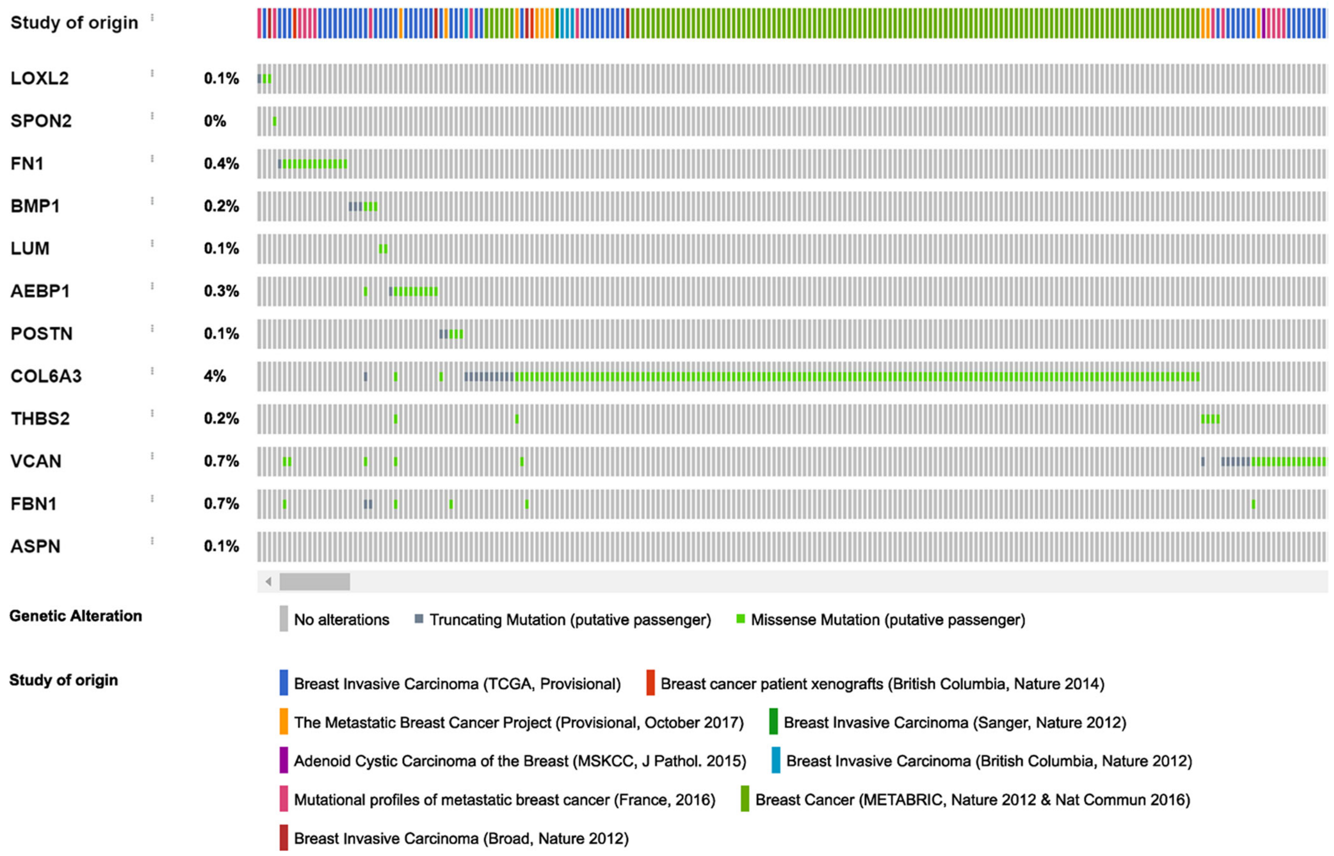

Supplementary Figure 1: *In silico* analysis of breast cancer databases. TCGA analysis for ECM-related gene expression via the cBioPortal database.
